# A Factor Produced by *Kaistia* sp. 32K Accelerated the Motility of *Methylobacterium* sp. ME121

**DOI:** 10.3390/biom10040618

**Published:** 2020-04-16

**Authors:** Yoshiaki Usui, Yuu Wakabayashi, Tetsu Shimizu, Yuhei O. Tahara, Makoto Miyata, Akira Nakamura, Masahiro Ito

**Affiliations:** 1 Graduate School of Life Sciences, Toyo University, Oura-gun, Gunma 374-0193, Japan; ysak4415@gmail.com (Y.U.); canonoftheend@gmail.com (Y.W.); 2 Faculty of Life and Environmental Sciences, and Microbiology Research Center for Sustainability (MiCS), University of Tsukuba, Tsukuba, Ibaraki 305-8572, Japan; sakuratettyan@hotmail.com (T.S.); nakamura.akira.fm@u.tsukuba.ac.jp (A.N.); 3 Department of Biology, Graduate School of Science, Osaka City University, Osaka 558-8585, Japan; taharayuhei@gmail.com (Y.O.T.); miyata@sci.osaka-cu.ac.jp (M.M.); 4 The OCU Advanced Research Institute for Natural Science and Technology (OCARINA), Osaka City University, Osaka 558-8585, Japan; 5 Bio-Nano Electronics Research Centre, Toyo University, Kawagoe, Saitama 350-8585, Japan

**Keywords:** symbiosis, coculture, motility, *Methylobacterium*, *Kaistia*

## Abstract

Motile *Methylobacterium* sp. ME121 and non-motile *Kaistia* sp. 32K were isolated from the same soil sample. Interestingly, ME121 was significantly more motile in the coculture of ME121 and 32K than in the monoculture of ME121. This advanced motility of ME121 was also observed in the 32K culture supernatant. A swimming acceleration factor, which we named the K factor, was identified in the 32K culture supernatant, purified, characterized as an extracellular polysaccharide (5–10 kDa), and precipitated with 70% ethanol. These results suggest the possibility that the K factor was directly or indirectly sensed by the flagellar stator, accelerating the flagellar rotation of ME121. To the best of our knowledge, no reports describing an acceleration in motility due to coculture with two or more types of bacteria have been published. We propose a mechanism by which the increase in rotational force of the ME121 flagellar motor is caused by the introduction of the additional stator into the motor by the K factor.

## 1. Introduction

Microorganisms often establish symbiotic relationships with other species. Because bacteria share their habitat with other microorganisms, increasing studies on cocultivation by intentionally mixing and culturing different bacteria have been conducted. For example, Olson et al. reported that the interspecific interaction between *Candida albicans* and *Candida glabrata* increased biofilm formation and virulence-related gene expression in a composition-dependent manner [1]. Onaka et al. reported that coculturing actinomycetes and bacteria produces antibiotics that are not produced under monoculture [2]. Thus, coculture studies are expected to reveal new bacterial properties not observed during monoculture. To date, cocultivation studies have mainly focused on growth, probiotics, and metabolic products [3,4,5].

*Methylobacterium* sp. ME121 and *Kaistia* sp. 32K were isolated from the same soil sample during a search for bacteria capable of assimilating L-glucose [6,7]. ME121 has a unipolar flagellum and is motile (Supplementary Figure S1A, Movies S1 and S2), whereas 32K has no flagellum and is thus not motile (Supplementary Figure S1B). We accidentally discovered that the swimming speeds of ME121 grown in a ME121-32K coculture showed a significantly accelerated motility compared with that in an ME121 monoculture. Nakamura et al. reported a deceleration in bacterial swimming speed upon the cocultivation of the lactose-fermenting bacteria *Lactococcus lactis* subsp. lactis and *Salmonella enterica serovar Typhimurium* [5]. The motility of *Salmonella* was either decreased or lost due to acidic substances produced by the lactic acid bacteria. Another study demonstrated the enhancement of bacterial motility in an *Escherichia coli* monoculture due to the production of an attractant [8]. However, no report has been published describing an acceleration in motility by a coculture with two or more types of bacteria.

This study was initiated based on the observation that the swimming speed of ME121 increased in a mixed culture with 32K. Therefore, we attempted to elucidate the mechanism underlying this advanced motility by investigating the properties of the substances derived from the 32K culture supernatant.

## 2. Materials and Methods

### 2.1. Bacterial Strains and Growth Media

*Methylobacterium* sp. ME121 and *Kaistia* sp. 32K were used in this study. 

For ME121, a Met medium (10.0 g of peptone, 2.0 g of yeast extract, 1.0 g of MgSO_4_, and 5 mL of methanol per liter) was used for the preculture, whereas for 32K, an LM medium (10.0 g of tryptone, 5.0 g of yeast extract, and 1.0 g of d-mannitol per liter) was used for the preculture. Methanol was filter-sterilized with Millex-LG filters (Merck KGaA, Darmstadt, Germany, pore size: 0.2 μm). 

A d-glucose synthetic medium (1.07 g of NH_4_Cl, 0.81 g of MgCl_2_, 0.75 g of KCl, 1.74 g of KH_2_PO_4_, 1.36 g of K_2_HPO_4_, 2 mL of Hutner’s trace elements, and 0.90 g of d-glucose per liter) was used for the monoculture and the coculture. Hutner’s trace elements were prepared by dissolving 22.0 g of ZnSO_4_·7H_2_O, 11.4 g of H_3_BO_3_, 5.06 g of MnCl_2_·7H_2_O, 1.16 g of CoCl_2_·5H_2_O, 1.57 g of (NH_4_)_6_Mo_7_O_24_·4H_2_O, and 1.57 g of FeSO_4_·7H_2_O in 500 mL of sterile water. EDTA·2Na (50 g) was dissolved while warming 300 mL of Milli-Q water. The pH was adjusted in the 6.5–6.8 range with KOH after the addition of each component, and its final volume was adjusted to 1 L. The solution was stored at 4 °C for approximately two weeks until its color changed from light green to purple red. It was then sterilized by filtration and used for the d-glucose synthetic medium. *E. coli* W3110 was used as the control for the tethered cell assay of ME121.

### 2.2. Monoculture and Coculture Conditions

The cells of ME121 and 32K were cultured in 10 mL of the Met and LM media (28 °C, 300 rpm, 48 h), respectively, washed with saline, and suspended in 2 mL of the d-glucose synthetic medium.

For the monoculture, the cells were inoculated into Φ24-mm test tubes containing 10 mL of the d-glucose synthetic medium to ensure an initial optical density (OD_600_) of 0.08, and then they were cultured (28 °C, 300 rpm). For the coculture, 5 mL of each bacterial suspension (OD_600_ = 0.08) were mixed in the same test tube and cultured (28 °C, 300 rpm).

A tethered cell assay was conducted for bacterial flagellar rotation analysis, in which a single colony of *E. coli* W3110 was cultured in 2 mL of an LB medium (30 °C, 200 rpm, 14 h). *E. coli* W3110 was precultured in 2 mL of the LB medium (OD_600_ = 0.01; 30 °C, 200 rpm, 7 h). 

### 2.3. Combined Cultures Established in a Beppu Flask

**Figure 1:**
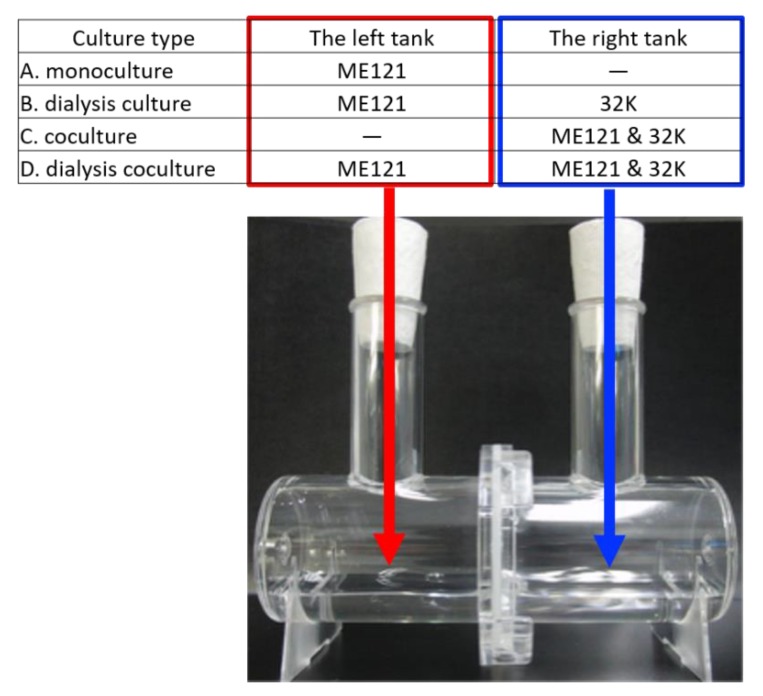
Establishing combined cultures in Beppu flask. The combination of inocula in the culture tanks separated by a membrane filter (Supor 200 hydrophilic polyether sulfone; pore size: 0.2 μm; diameter: 44 mm) was (A) monoculture, (B) dialysis culture, (C) coculture and (D) dialysis coculture. The optical density (OD_600_) measurement on the left tank in the coculture (C) was not performed.

In this paper, “coculture” refers to mixed cultures of two types of bacteria, whereas “dialysis culture” refers to separate cultures of two types of bacteria established in a Beppu flask [9] (Nihon Pall Corporation, Tokyo, Japan) with two tanks partitioned by a membrane filter (pore size: 0.2 μm), as shown in Figure 1. In this “dialysis coculture” the cultures are established in a Beppu flask with one tank inoculated with the pure culture of ME121 and the other tank inoculated with the mixed culture of ME121 along with 32K, as shown in Figure 1. 

Briefly, 10 mL of the synthetic d-glucose medium or cells suspended in the same medium were inoculated into both culture tanks (OD_600_ = 0.08) and cultured (28 °C, 300 rpm). For the coculture of ME121 and 32K, 5 mL of each bacterial suspension (OD_600_ = 0.08) were inoculated in the d-glucose synthetic medium and placed in the same culture tanks. The OD_600_ of the left culture tank in Figure 1 was measured every 24 h. 

### 2.4. ME121 Culture in 32K Culture Supernatant

To evaluate the growth and swimming speed of ME121 cultured in the 32K culture supernatant, the cells of ME121 cultured in 10 mL of the Met medium (28 °C, 300 rpm, 48 h) were washed with saline and suspended in 2 mL each of the d-glucose synthetic medium and the 32K culture supernatant. Each was suspended into a test tube containing 10 mL of the d-glucose synthetic medium and the 32K culture supernatant (OD_600_ = 0.08), then cultured (28 °C, 300 rpm, 72 h). The OD_600_ of each culture was measured every 24 h.

### 2.5. Motility Assay

Bacterial motility was observed under a dark-field microscope (Leica DMRE; Leica geosystem, Tokyo Japan) while maintaining the culture solution at 28 or 32 °C on a microscope stage (Type: MP-2000, Leica) [10]. The swimming speed of ME121 was almost the same when the plate temperature was 28 or 32 °C. We recorded a video of the observed movements using a digital color camera (Leica DF310 FX). The speed of each swimming cell was calculated using 2D movement measurement capture 2D-PTV software (Digimo, Tokyo, Japan) and the captured movie. Three independent experiments were conducted, and at least 60 bacterial cells were measured. Statistical analysis was performed by a Microsoft Excel *t*-test.

### 2.6. Swimming Speed of ME121 in the 32K Culture Supernatant

A single colony of ME121 was cultured in 10 mL of the Met medium (28 °C, 300 rpm, 48 h), and 50 µL of the preculture broth was inoculated into a test tube containing 10 mL of the Met medium and then cultured (28 °C, 300 rpm, 24 h). The culture broth (1 mL) was centrifuged (room temperature, 9100× *g*, 5 min). The cells of ME121 were resuspended in 1 mL of the swimming assay medium.

Five types of swimming assay media were tested: (i) the Met medium, (ii) the synthetic d-glucose medium, (iii) the synthetic Met medium, (iv) the carbon-free synthetic medium, and (v) the 32K culture supernatant. The synthetic Met medium contained 5 mL of methanol instead of 0.9 g of d-glucose per liter, as was in the d-glucose medium. The carbon-free synthetic medium was used to remove d-glucose from the d-glucose medium. 

Immediately after suspension, the microbial cells were kept at 28 or 32 °C on a glass heater; the motility of the bacteria was observed with a dark-field microscope, and their appearance was video-recorded. Three independent experiments were conducted with at least 100 bacterial cells.

### 2.7. Various Treatments of the 32K Culture Supernatant

#### 2.7.1. Heat Treatment

The 32K culture supernatant was heated at 121 °C for 40 min using an autoclave, after which it was returned to room temperature.

#### 2.7.2. Lipid Removal Treatment

A 10 mL aliquot of the 32K culture supernatant was added to 20 mL of a chloroform/ethanol mixture (2:1) and mixed thoroughly. After separating the aqueous layer, ethanol was removed with a rotary evaporator (N-1100, Eyela, Tokyo, Japan). Distillation was performed in an eggplant flask placed in a 37 °C water bath for 20 min. Sterile water was added to the aqueous layer until a final volume of 10 mL.

#### 2.7.3. Protein Removal Treatment with an Enzyme

The amount of protein contained in the 32K culture supernatant was calculated using the Lowry method. Proteinase K (Merck Millipore) was added to the 32K culture supernatant to a final concentration of 0.090 Anson units/mL and incubated at 37 °C for 1 h. After incubation, the enzyme was kept at 75 °C for 10 min for inactivation, and the 32K culture supernatant was returned to room temperature.

#### 2.7.4. Ethanol Precipitation

The 32K culture supernatant (50 mL) was dispensed in an eggplant flask and frozen at −30 °C. The frozen 32K culture supernatant was lyophilized overnight with a freeze-drier (VD-250R; Taitec Co., Ltd., Japan) and then dissolved in 5 mL of sterilized water. The concentrated 32K culture supernatant (5 mL) was desalted by dialysis (4 °C, 24 h) with a Spectra/Por 6 instrument (Spectrum Laboratories; diameter: 11.5 mm; membrane material: standard regenerated cellulose membrane [standard RC membrane], molecular weight: 3500 Da cutoff). Using 1 L of Milli-Q water, the external solution was changed three times. The desalted 32K culture supernatant (approximately 6 mL) was lyophilized overnight and then dissolved in 6 mL of sterilized water. To the suspension, 14 mL of 99.5% ethanol was added, and the mixture was allowed to stand overnight at −30 °C. The sample was centrifuged (4 °C, 13,000× *g*, 1 h); the supernatant (nonpolar fraction) was separated from the precipitate (polar fraction), and the former was discarded. The polar fraction was dissolved in 10 mL of sterilized water, and the ethanol was removed with a rotary evaporator. The whole polar fraction (approximately 10 mL) was frozen at −30 °C, lyophilized overnight, and dissolved in 50 mL of sterilized water.

#### 2.7.5. Dialysis

The 32K culture supernatant (5 mL) was inoculated at 4 °C for 24 h using the Spectra/Por® 6 instrument (Spectrum Laboratories, Rancho Dominguez, CA, USA; diameter: 11.5 mm; membrane material: standard RC membrane; molecular weight: 3500 Da cutoff). For the external solution, a carbon-free medium (1 L) was used and dialyzed by exchanging the external solution three times.

#### 2.7.6. Ultrafiltration

The 32K culture supernatant (5 mL) was added to a centrifugal filtration filter and centrifuged (4 °C, 2200× *g*, 90 min; RLX-105, Tomy Seiko). This solution was used for the motility assay. The following membranes were used as centrifugal filtration filters: an Amicon Ultra-15 centrifugal filter unit (Merck Millipore; membrane material: ultra-cell regenerated cellulose membrane; nominal molecular weight limit: 10,000 Da) and the VIVA SPIN 15R (Sartorius; membrane material: Hydrosart; nominal molecular weight limit: 5000 Da).

### 2.8. Swimming Speed of ME121 at Various pH Values

ME121 was cultured in 10 mL of the Met medium. After harvesting, the cells were resuspended in the d-glucose synthetic medium (pH 5.0, 5.5, or 6.0), after which the swimming speed was recorded as described previously. The pH was adjusted with 6 N HCl. Three independent experiments were conducted with at least 100 bacterial cells.

### 2.9. Preparation of the 32K Culture Supernatant

32K was cultured in 100 mL of the LM medium (28 °C, 200 rpm, 48 h) and centrifuged (4 °C, 9100× *g*, 5 min). The cells were resuspended in 50 mL of saline and centrifuged (4 °C, 9100× *g*, 5 min). The pellet was inoculated in a 5 L jar fermenter (BMS-05; Able Co., Ltd., Tokyo, Japan) containing 2.9 L of the d-glucose synthetic medium to achieve an initial OD_600_ = 0.08, and then it was cultured at 28 °C using a stirring blade rotating at 750 rpm and an aeration rate of 3 L/min. After the 32K growth reached the stationary phase, the culture supernatant was harvested by centrifugation (4 °C, 14,000× *g*, 30 min). The culture supernatant (2.5 L) was sterilized using a Nalgene Rapid-Flow Polyethersulfone (PES) membrane filter unit (pore diameter: 0.2 μm; Thermo Fisher Scientific).

### 2.10. Refining of the Motility-Accelerating Factor

Bacterial motility was simultaneously observed with growth measurements using a dark-field microscope at 32 °C on a glass heater every 12 h until ME121 lost its motility. Three independent experiments were conducted with at least 60 bacterial cells.

### 2.11. Preparation of the K Factor

The 32K culture supernatant (50 mL) was dialyzed overnight against the Milli-Q water (molecular weight: 3500 Da cutoff, 4 °C). The desalted culture supernatant was lyophilized overnight in a freeze-dryer (EYELA FDU-2200). After dissolving the dried sample in 6 mL of sterilized water, ethanol was added to a final concentration of 70%, and the mixture was allowed to stand at −30 °C overnight. Next, the precipitate and the supernatant were separated by centrifugation (4 °C, 13,000× *g*, 1 h; Tomy Seiko MX-305, Tokyo). Ethanol was removed in the supernatant using rotary evaporation (37 °C, 10 min; EYELA, Type N-1210B). The precipitated fraction was resuspended in 10 mL of sterilized water, and ethanol was removed as described above. The suspension was again lyophilized overnight, and the lyophilized powder was resuspended in a 50 mM acetic acid/NaOH buffer solution (pH 5.0). The solution was loaded on a DE-52 DEAE-cellulose column (2.5 × 50 cm; GE Healthcare Japan, Hino, Japan) that had been equilibrated with a 50 mM acetic acid/NaOH buffer (pH 5.0) [11]. The column was washed with 500 mL of the buffer at a flow rate of 110 mL/h. The column was eluted at the same rate with 250 mL of a buffer containing 0.2 M NaCl, followed by a linear gradient elution from 0.2 to 0.6 M NaCl in the buffer (700 mL) at a flow rate of 60 mL/h. Fractions (12 mL) containing neutral sugars were pooled, dialyzed against deionized water, and concentrated in a rotary evaporator (43 °C). Neutral sugars were determined using the anthrone reagent method with glucose as a reference [12].

### 2.12. Analysis of the Monosaccharide Composition of the K Factor

Trifluoroacetic acid (100 μL; 4 M) was added to the K factor (lyophilized product; 1.28 mg), and the mixture was incubated at 100 °C for 3 h. The hydrolysate was dried, solidified, dissolved in 100 μL of ultrapure water, and centrifuged (4 °C, 10,000 g, 10 min). The supernatant (50 μL) was recovered. Then, 50 μL of the supernatant was diluted 10-fold with ultrapure water, *N*-acetylated using acetic anhydride, and subjected to fluorescence labeling with the *p*-aminobenzoic acid ethyl ester (ABEE) reagent [13]. Thereafter, the monosaccharides labeled with fluorescence were recovered from the water layer by chloroform extraction and used for analysis. The analytical conditions were as follows: boric acid buffer/acetonitrile; flow rate, 0.5 mL/min; detection, fluorescence (Ex: 305 nm; Em: 360 nm); BioAssist EZ (Tosoh, Tokyo, Japan); and column, PN-PAK C18 (3.0 × 75 mm).

### 2.13. Flagellar Motor Rotation

The ME121 preculture broth (50 μL) was inoculated into a test tube containing 10 mL of the Met medium and was cultured (28 °C, 300 rpm, 24 h). This culture broth (1 mL) was subjected to shearing 20 times using a 1 mL syringe and injection needle. The ME121 culture medium (40 μL) was poured between a glass slide (S 1225; Matsunami Glass Industry Co., Ltd., Osaka, Japan) and a glass cover slip (Thickness No. 1; Matsunami Glass Industry Co., Ltd.), so that the side of the cover slip faced downward. It was allowed to stand at 28 °C for 20 min, and the flagella were then adsorbed onto the side of the glass cover slip. The d-glucose synthetic medium (40 μL) was poured between the glass cover slip and the glass slide to wash away the unadsorbed bacterial cells. This was performed twice. Subsequently, cell rotation with flagellar rotation was observed using a dark-field microscope and was video recorded. After recording, the solution was exchanged into the 32K culture supernatant in the same manner. This operation was also performed twice, and cell rotation was video-recorded as described above. The rotation speed per second was calculated using the ImageJ version 1.50i software (National Institutes of Health). At least three independent experiments were conducted, and the rotational speed of at least 100 cells was measured. The same operation was repeated for *E. coli* W3110, which served as the control.

### 2.14. Fluorescent Staining of the Flagella

The fluorescent staining of flagella was performed according to the method of Kinoshita et al. with slight modifications [14]. Briefly, the ME121 culture in the Met medium (1 mL) was transferred into a 1.5 mL tube and centrifuged (24 °C, 9100× *g*, 3 min). The pellet was resuspended in 1 mL of a phosphate buffer (0.81 g of MgCl_2_, 1.36 g of KH_2_PO_4_, and 1.36 g of K_2_HPO_4_ per liter), centrifuged (24 °C, 9100× *g*, 3 min), and resuspended in 0.1 mL of the phosphate buffer. Cy *N*-Hydroxysuccinimide (NHS) ester monoreactive dye (GE Healthcare) was added to the bacterial suspension. Subsequently, dyeing was carried out at room temperature in the dark for 30 min to 1 h. Next, the suspension was centrifuged (24 °C, 9100× *g*, 3 min), and the pellet was suspended in 1 mL of the phosphate buffer to remove the excess fluorescent reagent. Thereafter, the suspension was centrifuged (24 °C, 9100× *g*, 3 min), and the pellet was resuspended in 50 μL of the d-glucose synthetic medium or the 32K culture supernatant. While maintaining the temperature at 32 °C with a glass heater, the swimming behavior of the bacteria was observed with a dark-field microscope. To each cell suspension, 50 μL of a 2% methylcellulose solution was added and mixed. The flagellar structure of the stained cells was observed under a phase-contrast fluorescence microscope. Cell images were acquired using the Leica DF310 FX camera. The pitch of each flagellar filament was analyzed using the ImageJ version 1.50i software. Three independent experiments were conducted with at least 100 bacterial cells.

### 2.15. Quick-Freeze, Deep-Etch Electron Microscopy

ME121 and 32K cell suspensions in the logarithmic growth phase were collected by centrifugation (room temperature, 8000× *g*, 5 min) and suspended in a buffer consisting of 10 mM HEPES (pH 7.6), 150 mM NaCl, and 1 mM MgCl_2_ to achieve a 20-fold higher cell density. The cell suspensions were mixed with a slurry that included mica flakes, placed on a piece of rabbit lung, and frozen with a CryoPress (Valiant Instruments, St. Louis, MO, USA) that was cooled by liquid helium [15]. The slurry was used to retain an appropriate amount of water before freezing. The specimens were fractured and etched for 15 min at −104 °C in a JFDV freeze-etching device (JEOL Ltd., Akishima, Japan) [16]. The exposed cells were rotary shadowed by platinum at an angle of 20 degrees to a 2 nm thickness and backed with carbon. The replicas were floated off on full-strength hydrofluoric acid, rinsed in water, cleaned with a commercial bleach, rinsed again in water, and picked up onto copper grids as described [17,18]. They were observed under a JEM-1010 transmission electron microscope (JEOL, Tokyo, Japan) at 80 kV equipped with a FastScan-F214 (T) Charge Coupled Device (CCD) camera (TVIPS, Gauting, Germany). 

## 3. Results

### 3.1. Growth and Swimming Speed of ME121 in Several Culture Conditions

This study was initiated by the accidental discovery that a coculture with the nonmotile bacterium 32K accelerated the swimming speed of the motile bacterium ME121. Therefore, we focused on the swimming acceleration product (herein termed as K factor) in the 32K culture supernatant, and we investigated its properties.

**Figure 2:**
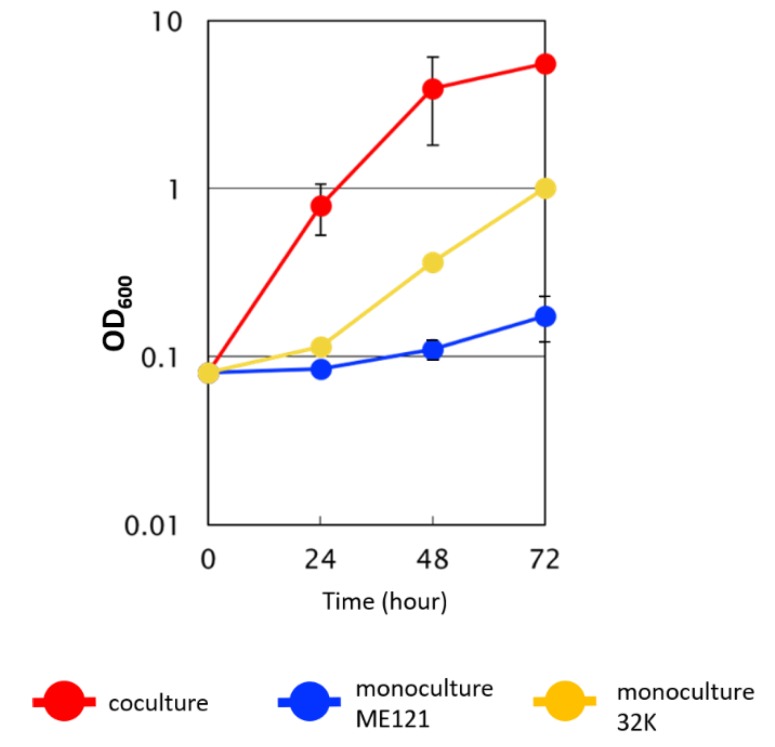
Growth curve of the monocultures of ME121 and of 32K and of coculture with ME121 and 32K in synthetic d-glucose medium in a test tube. Error bars indicate standard error.

**Figure 3:**
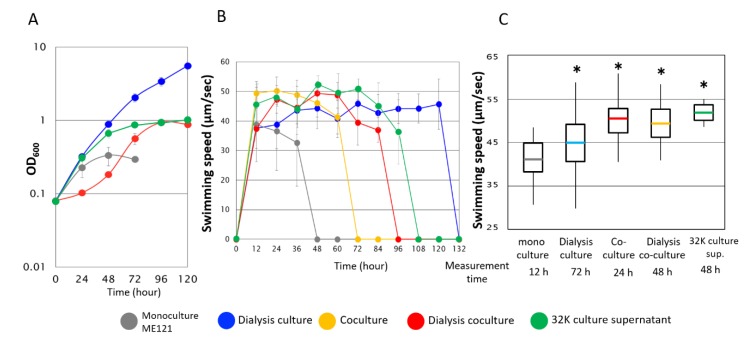
Growth and swimming speed of ME121 under several culture conditions in the synthetic d-glucose medium using a Beppu flask. (**A**) Growth curve, (**B**) swimming duration, and (**C**) swimming speed at exponential growth. In (**A**), the OD_600_ measurement on the left side of a Beppu flask during the coculture was not carried out. In (**C**), half of the values are within the box, and thick lines in the middle indicate average values. The line extending vertically indicates the remaining values, and the ends of each line indicate the maximum and the minimum values. * Significant difference from the d-glucose medium (*p* < 0.001). Statistical analysis was performed with a Microsoft Excel *t*-test. Values are expressed as the mean of three independent experiments, and the swimming speed of at least 60 cells was measured. Error bars indicate standard error.

When ME121 and 32K were separately cultured in the synthetic d-glucose medium, the growth of ME121 was not as good as when cocultured (Figure 2). When Beppu flasks were used, the swimming speed of ME121 in the logarithmic growth phase was the fastest among other growth phases under any other culture conditions (Figure 3). 

In the monoculture, ME121 reached the stationary phase in 48 h, while in contrast, growth was observed in the dialysis culture and the dialysis coculture beyond 48 h (Figure 3A). Significant growth in various cocultures, as well as prolonged swimming period and an accelerated swimming speed of ME121 (Figure 3 and Supplementary Movies S1 and S2) compared with the monoculture was observed. No motility was observed when ME121 reached the stationary phase in each culture condition. When the fastest swimming speeds during the logarithmic growth phase of ME121 were compared, significant differences in swimming speeds were observed between the monoculture and other cultures (Figure 3C). In the dialysis culture, the coculture, and the dialysis coculture, the swimming speeds of ME121 were faster than that in the monoculture.

To elucidate the mechanism of the accelerated swimming speed of ME121, we investigated whether the 32K supernatant accelerated the swimming speed of ME121. We found that swimming speed peaked in the logarithmic growth phase (Figure 3). Significant ME121 growth and motility duration were observed when the 32K culture supernatant was used instead of the synthetic d-glucose medium (Figure 3A,B). A significant acceleration in the swimming speed was also observed (Figure 3C and Supplementary Movies S3 and S4). 

**Figure 4:**
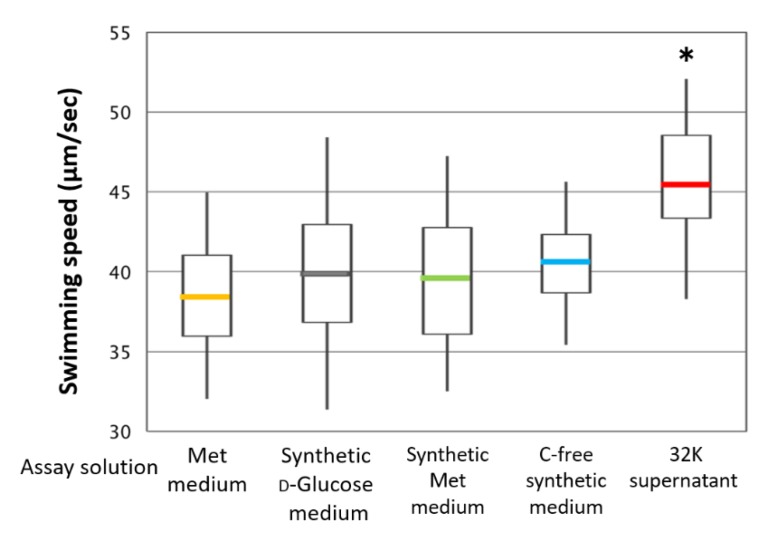
Swimming speed of ME121 in the 32K culture supernatant and other media. The distribution of the results of the motility test of the ME121 using the 32K culture supernatant was compared. The swimming speed of 100 cells was measured under each condition. The explanation of the box-and-whisker plot is shown in the legend of Figure 3. * Significant difference from the synthetic d-glucose medium (*p* < 0.001). Value are expressed as the mean of three independent experiments. For each growth, the synthetic d-glucose medium was used.

Suspensions in the Met medium, the synthetic d-glucose medium, the synthetic Met medium, or the Carbone (C)-free synthetic medium showed no increase in ME121 swimming speed; only the 32K culture supernatant significantly increased ME121’s swimming speed (Figure 4). Therefore, the K factor is not a metabolite of ME121. An enhancement in ME121 growth was also observed in the 32K culture supernatant. From this finding, we speculated that the K factor accelerated the growth of ME121, in addition to accelerating its swimming speed.

### 3.2. Analysis of the Monosaccharide Composition in the Ethanol-Precipitated Fraction of the 32K Culture Supernatant

**Figure 5:**
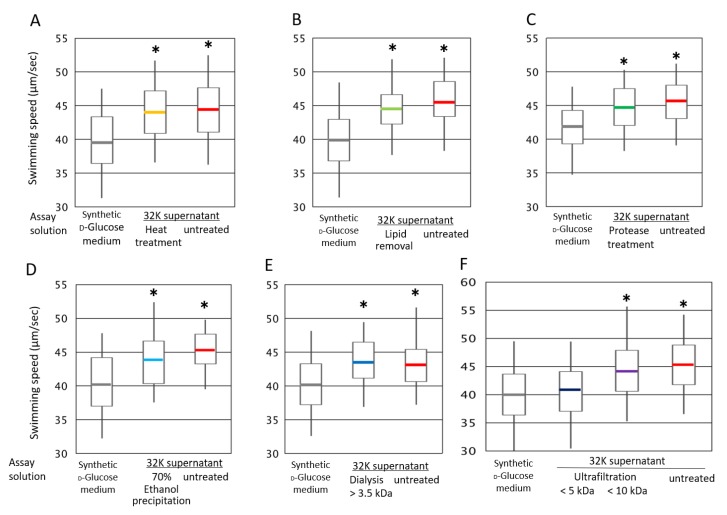
Swimming speed of ME121 in various treatments of the 32K culture supernatant: (**A**) heat, (**B**) lipid removal, (**C**) protein removal by enzyme, (**D**) ethanol precipitation, (**E**) dialysis, and (**F**) ultrafiltration. The variance of the results of the ME 121 motility test was compared. The explanation of the box-and-whisker plot is shown in the legend of Figure 3. * Significant difference from the synthetic d-glucose medium (*p* < 0.001). Values are expressed as the mean of three independent experiments, and the swimming speed of at least 100 cells was measured.

By analyzing the swimming speed of ME121 in the 32K culture supernatant solution that had been heated and treated with enzymes, the characteristics of the K factor of ME121 were estimated (Figure 5).

Heat treatment did not significantly change the swimming speed of ME121 (Figure 5A). This observation was similarly observed in treatments involving lipid and protein removal (Figure 5B,C). Therefore, the K factor is heat-stable and non-volatile, but it is neither a lipid or a substance affected by protease. Upon the ethanol treatment, a polar substance was precipitated. However, the aqueous solution of the precipitated polar substance had no significant effect on the ME121 swimming speed (Figure 5D). This suggested that the K factor is a polar substance precipitated in 70% ethanol.

The molecular weight of the K factor was estimated by dialysis and ultrafiltration. No significance differences in ME121 swimming speed were observed upon dialysis with a molecular weight cutoff of 3.5 kDa (Figure 5E). The molecular weight fraction of the 32K culture supernatant containing a substance of 10 kDa or less exerted the same effect on ME121 swimming speed as the 32K culture supernatant. However, when ME121 was suspended in the molecular weight fraction of the 32K culture supernatant that contained substances of 5 kDa or more, the swimming speed was almost the same as when ME121 was suspended in the synthetic d-glucose medium (Figure 5F). This result suggested that the molecular weight of the K factor is approximately 5–10 kDa.

### 3.3. Preparation of the K Factor

**Figure 6:**
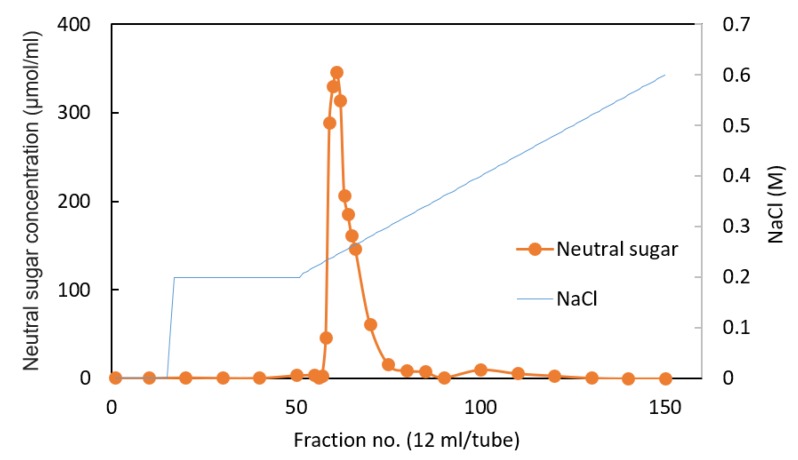
Purification of the ethanol precipitation of the 32K strain culture supernatant through a Diethylaminoethyl (DEAE)-cellulose column. Neutral sugars were quantified using the anthrone sulfate method. Fraction no. 58 to 80 were collected, desalted, freeze-dried, and subjected to the composition analysis indicated. Each fraction was collected in 12 mL portions. The concentration of NaCl was determined from the refractive index of the fractions.

As a result of the anion-exchange chromatography fractionation of the ethanol precipitation from the 32K culture supernatant using a DEAE cellulose column, substances containing neutral sugars with 0.23–0.32 M NaCl were eluted (Figure 6). 

Fractions 58 to 80, containing the highest neutral sugar concentration, were collected, desalted, lyophilized, and subjected to monosaccharide composition analysis.

### 3.4. Analysis of Monosaccharide Composition in the Ethanol-Precipitated Fraction (K Factor) of the 32K Culture Supernatant

The fluorescent pre-labeling method was used to analyze the monosaccharide composition (Table 1, Supplementary Figure S2). The neutral sugars glucose and galactose were present at a ratio of approximately 1:1 and accounted for approximately 55% of the total K factor. Uronic acids and amino sugars were not detected. Therefore, the K factor was presumed to be a kind of extracellular polysaccharide (EPS) composed of neutral sugars.

### 3.5. Elucidation of the ME121 Motility-Accelerating Mechanism of the K Factor

**Figure 7:**
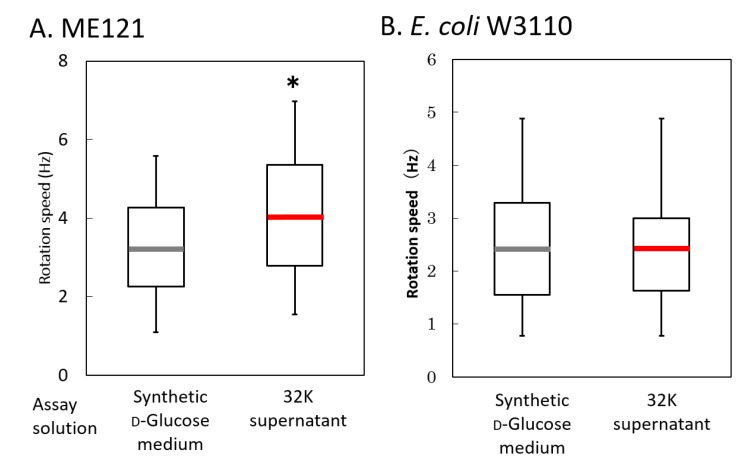
Rotation measurement of the flagellar motor using a tethered cell assay in ME121 (**A**) and W3110 (**B**). The variance of the results of the rotational measurement experiment of the flagellar motor of ME121 was compared with W3110. The vertical axis shows rotation speed (Hz). The explanation of the box-and-whisker plot is shown in the legend of Figure 3. * Significant difference from the synthetic d-glucose medium (*p* < 0.001). Values are expressed as the mean of three independent experiments, and the swimming speed of at least 100 cells was measured.

To elucidate the ME121 motility-accelerating mechanism, the measurement of the rotation of the flagellar motor was performed using a tethered cell assay, along with measurement of the pitch of the flagellar fibers of ME121 (Figure 7). When ME121 was exposed to the 32K culture supernatant, the rotational speed of the flagellar motor increased by approximately 25% (Figure 7A). In W3110, the rotational speed of the flagellar motor was not affected by the 32K culture supernatant (Figure 7B). This suggested that the acceleration was caused by an increase in the rotational power of the flagellar motor and that the increase in the rotational force of the flagellar motor was specific to ME121. Increased motility was also observed immediately after suspending ME121 in the 32K culture supernatant. 

**Figure 8:**
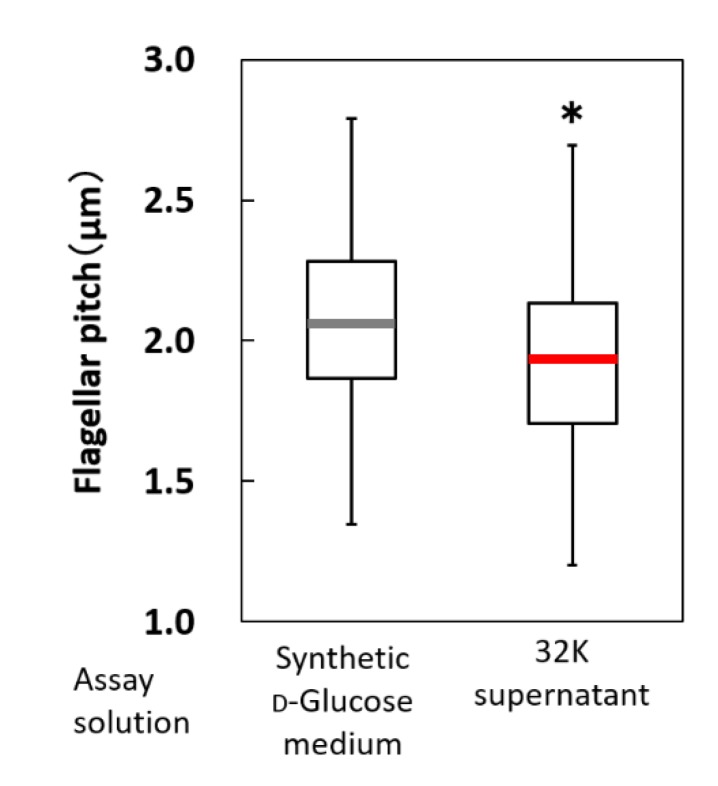
Flagellar pitch of strain ME121 with and without the 32K culture supernatant treatment. The variance of the measurement results of the pitch length of flagellar fiber of ME121 was compared with/without the 32K culture supernatant treatment. The explanation of the box-and-whisker plot is shown in the legend of Figure 3. * Significant difference from the synthetic d-glucose medium (*p* < 0.001). Values are expressed as the mean of three independent experiments, and the swimming speed of at least 100 cells was measured.

Next, the pitch of the flagellar fibers was determined in the presence or absence of the 32K culture supernatant. Specifically, fluorescent staining was used to investigate whether this pitch affected the swimming speed of ME121. The addition of the 32K culture supernatant also significantly accelerated the swimming speed of the stained flagellum ME121 (Supplementary Figure S3), indicating that fluorescent staining had no effect on the motility acceleration of ME121. The pitch of the flagellar fibers of ME121 was also measured (Figure 8). When the 32K culture supernatant was added, the pitch of the flagellar fibers of ME121 was shortened by approximately 10%.

**Figure 9:**
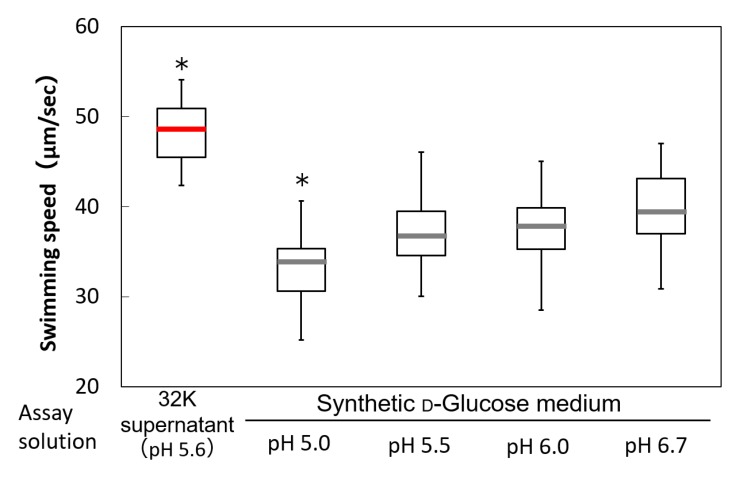
Swimming speed of ME121 using the 32K culture supernatant and the synthetic d-glucose medium at several pH values. The variance of the swimming speed measurement results of ME121 was compared using the 32K culture supernatant and the synthetic d-glucose medium with several pH values. The experimental method is similar to that outlined in Figure 8. The explanation of the box-and-whisker plot is shown in the legend of Figure 3. * Significant difference from the synthetic medium (pH 6.7; *p* < 0.001). Values are expressed as the mean of three independent experiments, and the swimming speed of at least 100 cells was measured.

The pH values of the synthetic d-glucose medium and the 32K culture supernatant were 6.74 and 5.56, respectively. To investigate the pH influence, the pH of the synthetic d-glucose medium was adjusted to match that of the 32K culture supernatant, after which the swimming speed of ME121 was analyzed (Figure 9). Even after the pH adjustment, the swimming speed of ME121 did not accelerate, suggesting that environmental pH was not involved in the accelerated motility of ME121.

Lastly, the elevated viscosity of the swimming environment did not increase the ME121 motility (Supplementary Figure S4). Thus, the acceleration of the ME121 swimming speed was not related to changes in environmental viscosity.

## 4. Discussion

### 4.1. 32K-Derived Products Stimulated the ME121 Growth and Swimming Speed

When ME121 and 32K were separately cultured in the synthetic d-glucose medium, growth was not as good as in the coculture. When using Beppu flasks, a significant improvement in growth, a prolonged swimming period, and an accelerated swimming speed were observed in all other culture conditions, compared with the ME121 monoculture. The growth of ME121 was initially slow in the dialysis culture, but its final OD_600_ was higher than that in the ME121 monoculture, and swimming duration was also prolonged for up to 84 h. In each culture condition, no swimming was observed once the stationary growth phase was reached. Plant-related *Methylobacterium* species and *Vibrio alginolyticus* release polar flagella after prolonged stationary culturing [19,20]. In *E. coli*, when nutrients are depleted, YcgR, a cyclic dimer guanosine monophosphate (c-di-GMP) binding protein, is activated, and YcgR and the flagellar switch complex proteins FliG and FliM interact to stop rotation without detaching the flagella [21,22]. Studies on the cessation of swimming of *Methylobacterium* species during prolonged stationary culturing and the mode of flagella are still poor and require for further investigation.

The swimming speed of ME121 was faster in the dialysis culture, the coculture, and the dialysis coculture than in the monoculture. In the dialysis coculture, the coculture in the right tank assimilated glucose in the medium and might have delayed the growth of the ME121 monoculture in the left tank. Therefore, the final OD_600_ reached by supplying the 32K-derived products was higher than that of the monoculture. It was concluded that these phenomena were due to 32K-derived products that stimulated the growth and swimming speed in the dialysis culture, the coculture, and the dialysis coculture conditions of ME121.

### 4.2. The Growth and Motility of ME121 Using the 32K Culture Supernatant

When the 32K culture supernatant was used as the medium, the growth of ME121 notably improved, and the swimming period was prolonged, indicating that the K factor was contained in the 32K culture supernatant. The increased swimming speed of ME121 was also observed immediately after suspension in the 32K culture supernatant. Therefore, the K factor was not metabolized by ME121. We expect that the 32K culture supernatant contains the K factor and growth factors promoting ME121. The growth factors may have been the same as the K factor.

### 4.3. The Motility of ME121 Using Treated Solutions (Heating, Dialysis, Enzyme Treatment, etc.) of the 32K Culture Supernatant

The K factor is a polar substance with a molecular weight of approximately 5–10 kDa. It is neither a lipid nor a protein cleaved by proteases. Therefore, we considered that the K factor is a sugar-containing complex or a sugar chain-modified protein.

### 4.4. Analysis of the Monosaccharide Composition of the Ethanol-Precipitated Fraction of the 32K Culture Supernatant

Because the neutral sugars glucose and galactose were present in a ratio of approximately 1:1 and accounted for approximately 55% of the K factor, the K factor was considered to be a kind of EPS composed of neutral sugars (Table 1). Demir and Salman reported that accelerated bacterial motility by attractants caused an increase in flagellar motor torque [23]. Bacteria are also known to move in the direction of attractants, such as amino acids and sugars [24]. Therefore, we report an unprecedented case of EPS-derived macromolecules driving the acceleration of bacterial swimming speed.

### 4.5. A Hypothesis on the Mechanism of Motion Acceleration of the Flagellar Motor of ME121 by the K Factor

The rotation speed of the ME121 flagellar motor was increased by approximately 25% when the 32K culture supernatant was added. However, in the case of W3110, an acceleration of the flagellar motor rotation speed of *E. coli* was not observed. This may be suggestive of the ME121 flagellar motor-specificity of the K factor. Because the acceleration of the rotation speed was immediately observed once the 32K culture supernatant was added, it is likely that the acceleration of ME121 swimming speed was due to an increase in the rotational force of the flagellar motor.

Furthermore, the addition of the 32K culture supernatant shortened the flagellar fiber pitch of ME121 by approximately 10%. Changes in flagellar pitch occur when environmental pH changes [25]. The pH of the synthetic d-glucose medium and the 32K culture supernatant were 6.74 and 5.56, respectively, suggesting that the short pitch of the flagellar filaments of ME121 may be affected by pH. However, no acceleration of the ME121 swimming speed was observed, indicating that differences in environmental pH are not involved in the acceleration of the ME121 motility.

From the abovementioned results, we proposed a mechanism underlying motility acceleration. The flagellar motor is considered to have a structure consisting of a stator and a rotor that is more dynamic than previously believed [26,27,28]. Upon activation, the stator is incorporated into the flagellar motor and generates a rotational driving force to rotate. *Bacillus subtilis* has two flagellar motor stators, MotAB and MotPS [29,30]. Though MotAB is more often used, *B. subtilis* uses MotPS, instead of MotAB, by recognizing the viscosity of the environment in the hydrophilic region of MotPS [31]. In our study, however, ME121 did not exhibit an improved motility even when the viscosity of the environment increased. Thus, the improvement in the ME121 swimming speed is not related to changes in viscosity. 

ME121 has a MotA/MotB stator (Accession no. ME121_1986 and ME121_1987). The attachment and detachment of the stator from the motor are dynamic and dependent on the load on the motor [32]. The maximum speed of the motor increases as additional stators are recruited to the motor. This is one of the proposed mechanisms by which the K factor increases ME121 swimming speed. In other words, the increase in the rotational force of the ME121 flagellum motor is caused by the introduction of the additional stator into the motor by the K factor. In other species, such as *Vibrio* spp., additional components (MotX and MotY) are known to increase bacterial swimming speed [33]. However, no such additional components in the flagellar motor of *Methylobacterium* spp. are known. No proteins homologous to MotX and MotY were identified in the ME121 genome sequence. We plan to elucidate this mechanism in the future by generating a ME121 mutant whose swimming speed is not increased by the K factor.

By recognizing the motility accelerating factor, ME121 was inferred to generate a stronger rotational driven force by incorporating more stators into the flagellar motor than usual. The coculture of ME121 and 32K improved the growth of both strains. 32K secreted the K factor by metabolizing nutrients in the medium. It was considered that the ME121 swimming speed was thereby accelerated. From the draft genome sequence of ME121, 33 methyl-accepting chemotaxis sensor (MCP) proteins involved in chemotaxis have been identified [6]. However, studies on chemotaxis and MCP in *Methylobacterium* spp. remain limited. It has been known that even nonmotile bacteria, such as 32K, have passive motility (colony spreading) [34].

In nature, bacteria often exist as agglomerations known as biofilms. Biofilm formation and bacterial motility are strongly related [35]. Generally, a motility defective mutant is related to poor biofilm formation [30]. Biofilms are formed by pathogenic bacteria on medical materials, such as intravascular indwelling catheters, artificial heart valves, and artificial joints, which cause infections [34]. In biofilm formation in cocultures using the opportunistic pathogens *Pseudomonas aeruginosa* and *Staphylococcus aureus*, the predominance of *P. aeruginosa* in the biofilm indicates that diguanylate cyclase is involved in matrix polysaccharide biosynthesis and its control from the early stage to the mature stage [36]. The modulation of the second messenger c-di-GMP levels is linked to bacterial swimming and biofilm formation [37]. 

We think that how ME121 senses the K factor, i.e., the mechanism of the accelerated motility, is associated with biofilm formation. Interestingly, when biofilm formation was studied using phylogenetically isolated *Methylobacterium*, biofilm formation in cocultures is enhanced compared with monocultures [38]. Therefore, biofilm formation in the coculture of ME121 and 32K strains should be further investigated. It would also be interesting to investigate whether the increase in the motility of *Methylobacterium* spp. is observed in the coculture of *Methylobacterium* spp. and *Kaistia* spp. isolated from different environments.

## 5. Conclusions

In this study, we suggested that the acceleration of ME121 motility is caused by the metabolites of 32K supernatant and not by the contact stimulation between cells. Our findings suggested that the K factor is an extracellular saccharide of 5–10 kDa produced by 32K and contains neutral sugars. We further inferred that motility was accelerated by the enhancement of the motor torque of the ME121 flagellar motor. We will promote the further elucidation of the swimming acceleration mechanism by the K factor. 

## Figures and Tables

**Table 1:** Monosaccharide composition of the ethanol-precipitated fraction of the 32K culture supernatant.

No.	Component Name	pmol	Per g of Sample	
			µmol	mg
1	Glucuronic acid	ND	ND	ND
2	Galacturonic acid	ND	ND	ND
3	Galactose	139	1.46 × 10^3^	263
4	Mannose	3.7	39	7.1
5	Glucose	145	1.53 × 10^3^	275
6	Arabinose	3.1	32	4.8
7	Ribose	ND	ND	ND
8	*N-acetyl-mannosamine*	ND	ND	ND
9	Xylose	1.2	13	1.9
10	*N-acetyl-glucosamine*	ND	ND	ND
11	Fucose	ND	ND	ND
12	Rhamnose	1.8	19	3.0
13	*N-acetyl-galactosamine*	ND	ND	ND

## Supplementary Materials

The following are available online at https://www.mdpi.com/2218-273X/10/4/618/s1, Figure S1: Quick-freeze deep-etch replica TEM imaging of a *Methylobacterium* sp. ME121 cell (A) and a *Kaistia* sp. 32K cell (B); Figure S2: Chromatogram of the monosaccharide composition in the ethanol precipitate fraction of the 32K culture supernatant; Figure S3: Swimming speed of ME121 in the 32K culture supernatant after fluorescence staining; Figure S4: Swimming speed of strain ME121 in a swimming assay buffer containing several concentrations of Ficoll 400; Video S1: (A) ME121 swimming in the monoculture of strain ME121 and (B) ME121 swimming in coculture of both strains ME121 and 32K strains; Video S2: (A) ME121 swimming in the synthetic d-glucose medium and (B) ME121 swimming in 32K culture supernatant.
