# Supplementary material for: A Factor Produced by Kaistia sp. 32K Accelerated the Motility of Methylobacterium sp. ME121
Source: Biomolecules. 2020 Apr 16;10(4):618. doi: 10.3390/biom10040618 (PMC7226442; doi:10.3390/biom10040618)
Supplement: Supplementary file 1 [file biomolecules-10-00618-s001.zip › Suporting data revise final.pptx]

## Slide 1
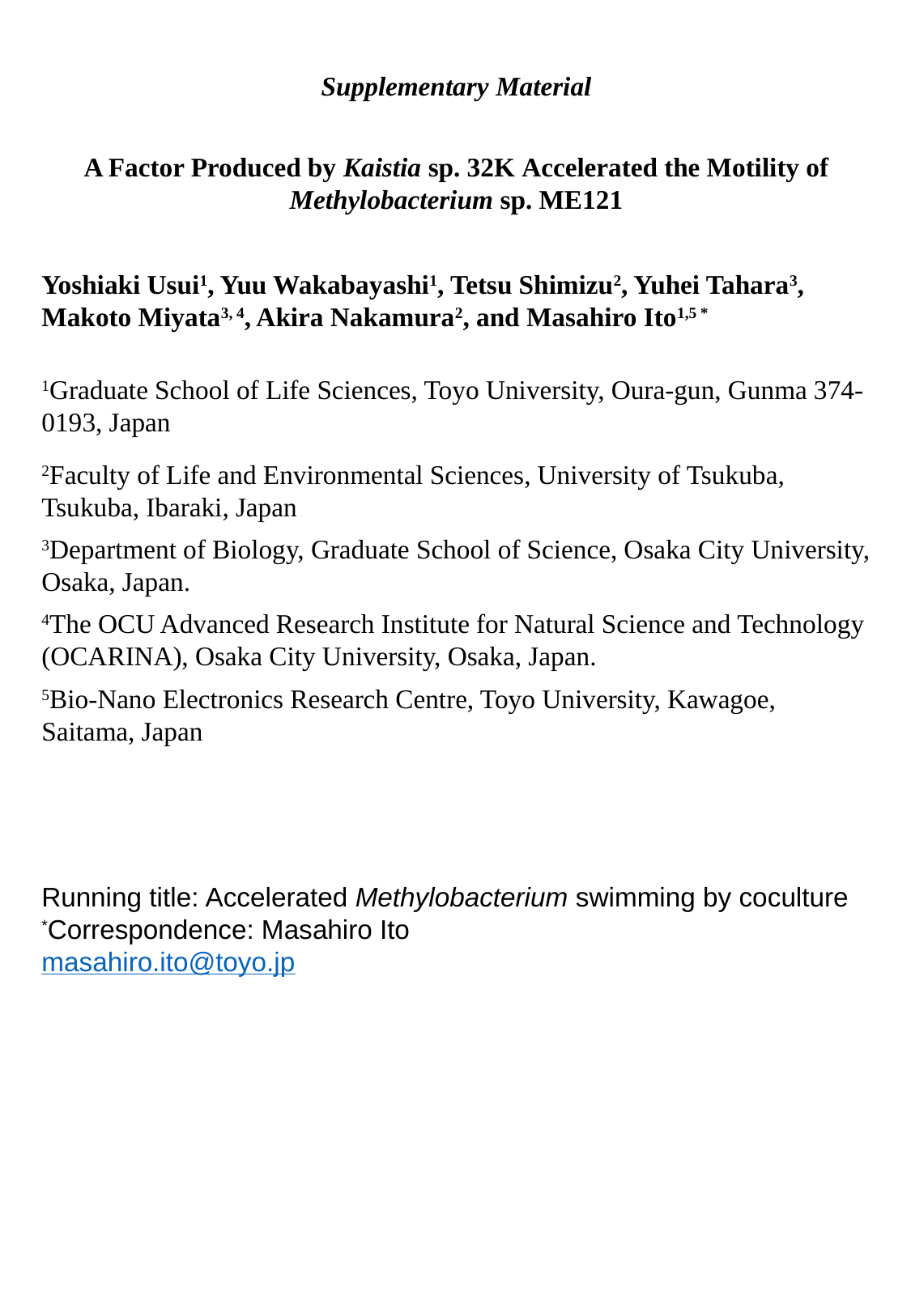

Supplementary Material
A Factor Produced by Kaistia sp. 32K Accelerated the Motility of Methylobacterium sp. ME121
Yoshiaki Usui1, Yuu Wakabayashi1, Tetsu Shimizu2, Yuhei Tahara3, Makoto Miyata3, 4, Akira Nakamura2, and Masahiro Ito1,5 *
1Graduate School of Life Sciences, Toyo University, Oura-gun, Gunma 374-0193, Japan
2Faculty of Life and Environmental Sciences, University of Tsukuba, Tsukuba, Ibaraki, Japan
3Department of Biology, Graduate School of Science, Osaka City University, Osaka, Japan.
4The OCU Advanced Research Institute for Natural Science and Technology (OCARINA), Osaka City University, Osaka, Japan.
5Bio-Nano Electronics Research Centre, Toyo University, Kawagoe, Saitama, Japan
Running title: Accelerated Methylobacterium swimming by coculture
*Correspondence: Masahiro Ito
masahiro.ito@toyo.jp

## Slide 2
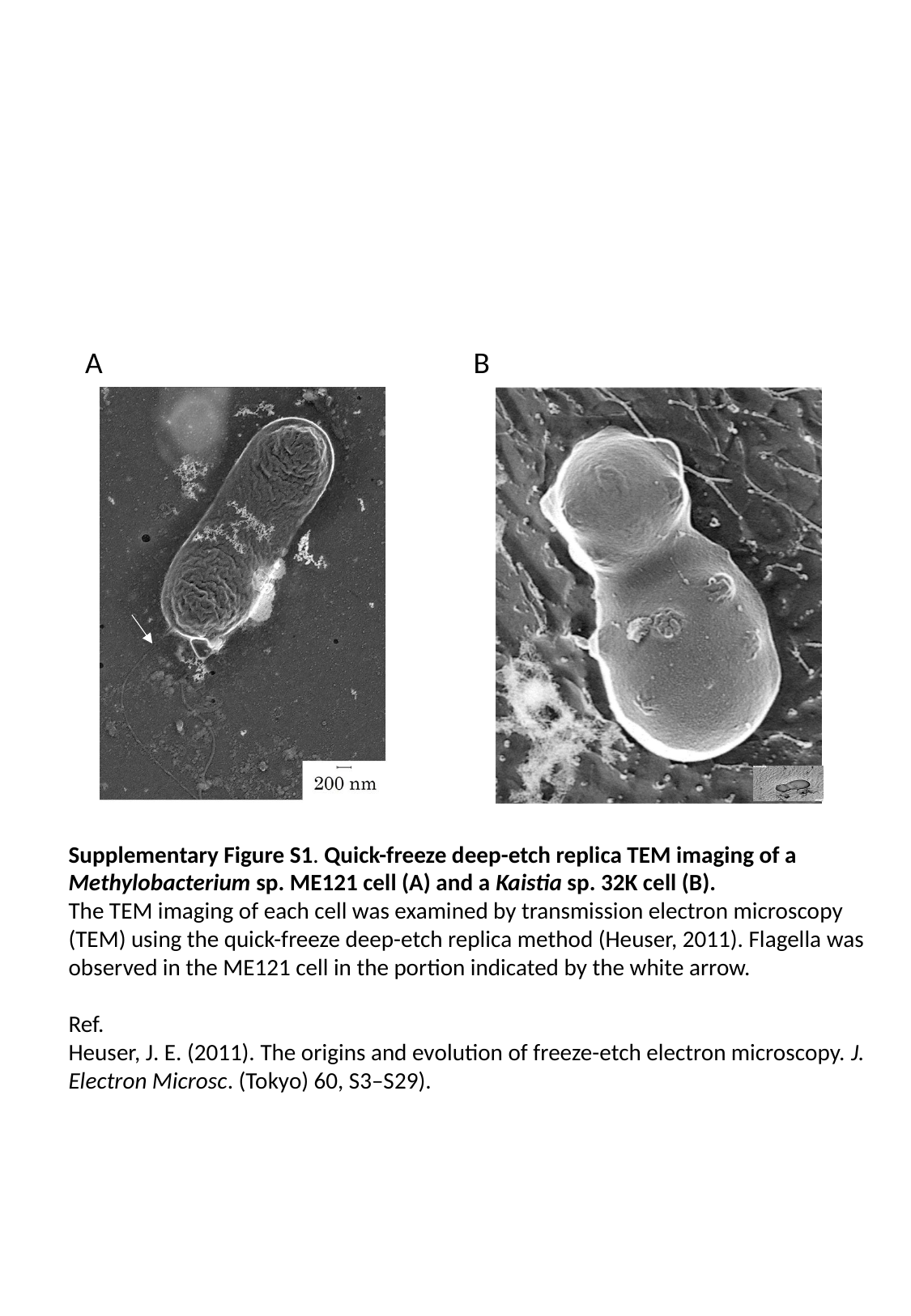

A B
Supplementary Figure S1. Quick-freeze deep-etch replica TEM imaging of a Methylobacterium sp. ME121 cell (A) and a Kaistia sp. 32K cell (B).
The TEM imaging of each cell was examined by transmission electron microscopy (TEM) using the quick-freeze deep-etch replica method (Heuser, 2011). Flagella was observed in the ME121 cell in the portion indicated by the white arrow.
Ref.
Heuser, J. E. (2011). The origins and evolution of freeze-etch electron microscopy. J. Electron Microsc. (Tokyo) 60, S3–S29).

## Slide 3
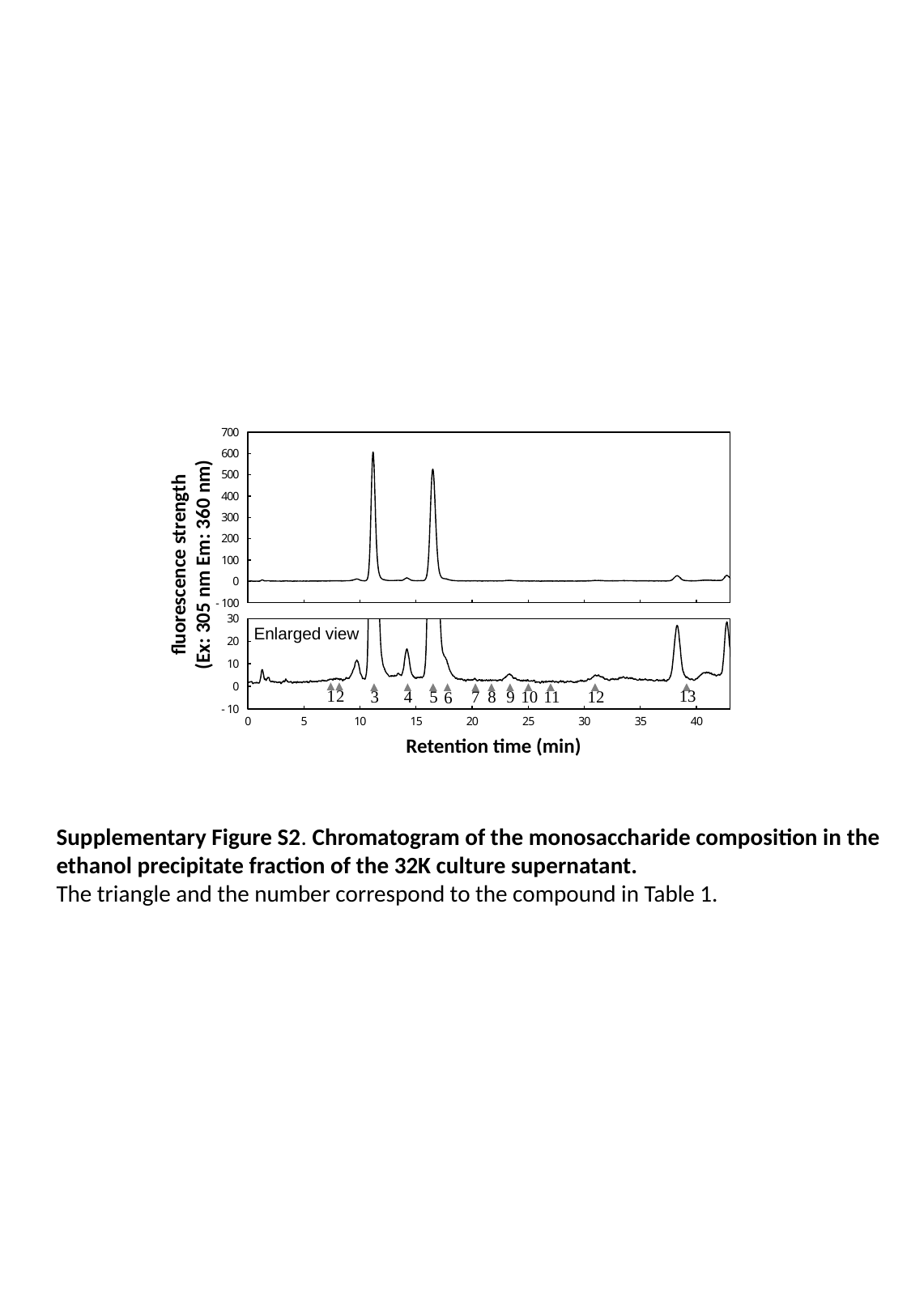

fluorescence strength
(Ex: 305 nm Em: 360 nm)
Enlarged view
1
2
13
3
4
5
7
8
9
10
11
12
6
Retention time (min)
Supplementary Figure S2. Chromatogram of the monosaccharide composition in the ethanol precipitate fraction of the 32K culture supernatant.
The triangle and the number correspond to the compound in Table 1.

## Slide 4
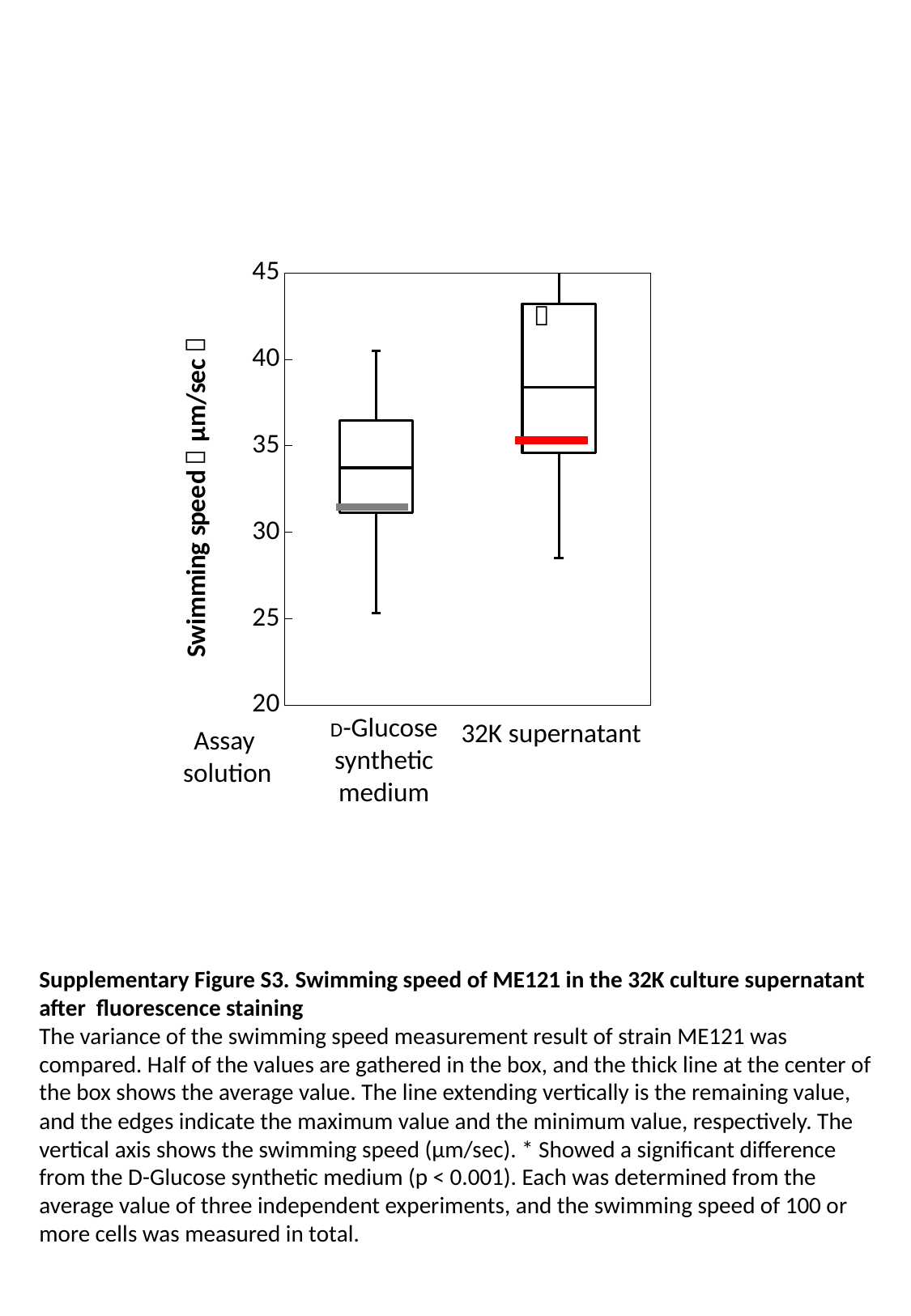

### Chart
| Category | | | |
|---|---|---|---|
| Glc medium | 31.1447619047619 | 2.5894077166669973 | 2.7306847475031404 |
| 32K supernatant | 34.58848484848484 | 3.814367793721239 | 4.815118372286683 |
＊
D-Glucose
synthetic medium
32K supernatant
Assay
solution
Supplementary Figure S3. Swimming speed of ME121 in the 32K culture supernatant after fluorescence staining
The variance of the swimming speed measurement result of strain ME121 was compared. Half of the values are gathered in the box, and the thick line at the center of the box shows the average value. The line extending vertically is the remaining value, and the edges indicate the maximum value and the minimum value, respectively. The vertical axis shows the swimming speed (μm/sec). * Showed a significant difference from the D-Glucose synthetic medium (p < 0.001). Each was determined from the average value of three independent experiments, and the swimming speed of 100 or more cells was measured in total.

## Slide 5
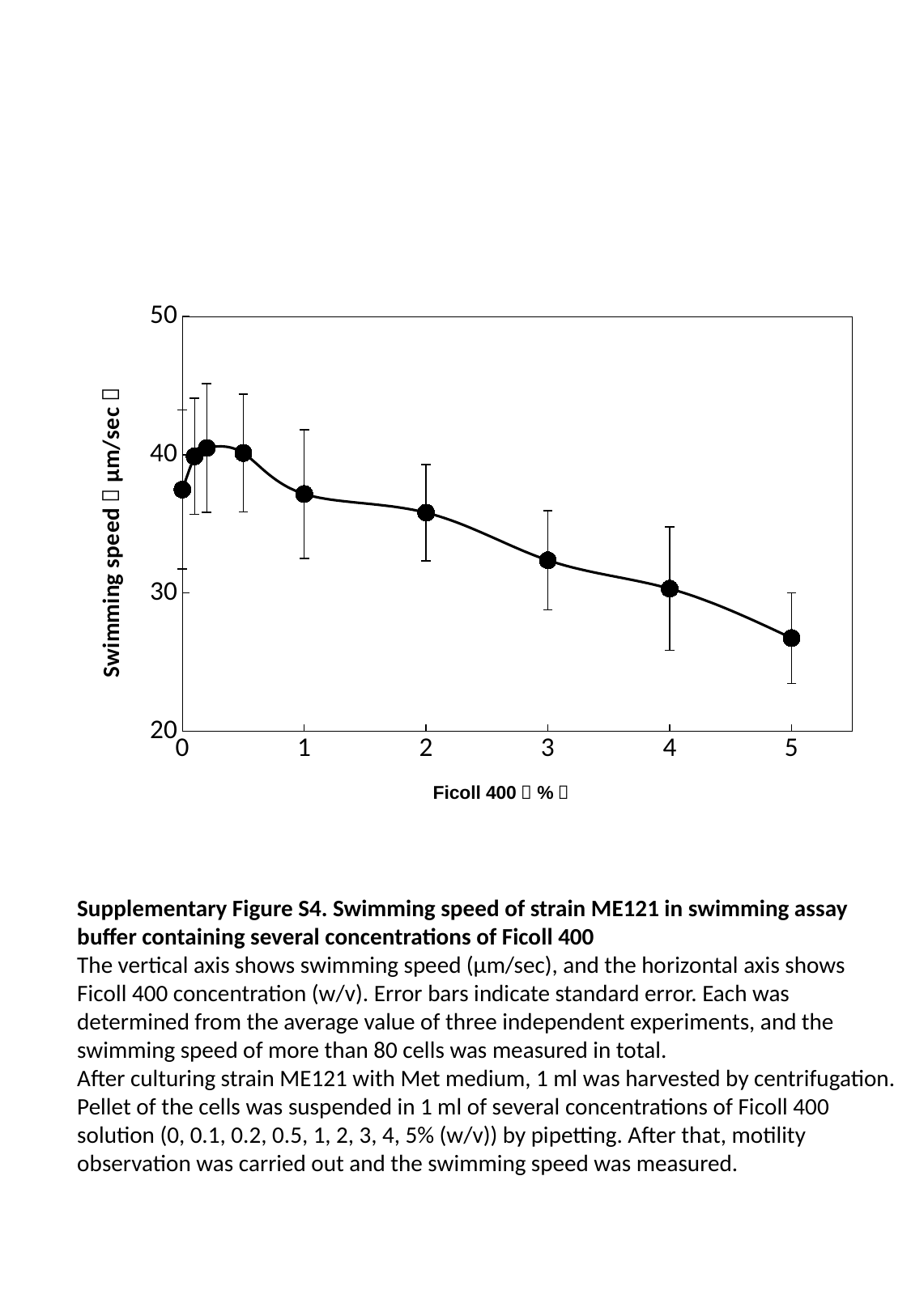

### Chart
| Category | |
|---|---|Supplementary Figure S4. Swimming speed of strain ME121 in swimming assay buffer containing several concentrations of Ficoll 400
The vertical axis shows swimming speed (μm/sec), and the horizontal axis shows Ficoll 400 concentration (w/v). Error bars indicate standard error. Each was determined from the average value of three independent experiments, and the swimming speed of more than 80 cells was measured in total.
After culturing strain ME121 with Met medium, 1 ml was harvested by centrifugation. Pellet of the cells was suspended in 1 ml of several concentrations of Ficoll 400 solution (0, 0.1, 0.2, 0.5, 1, 2, 3, 4, 5% (w/v)) by pipetting. After that, motility observation was carried out and the swimming speed was measured.
