# Supplementary material for: A Factor Produced by Kaistia sp. 32K Accelerated the Motility of Methylobacterium sp. ME121
Source: Biomolecules. 2020 Apr 16;10(4):618. doi: 10.3390/biom10040618 (PMC7226442; doi:10.3390/biom10040618)
Supplement: Supplementary file 1 [file biomolecules-10-00618-s001.zip › Supporting Video S1 and S2.pptx]

## Slide 1
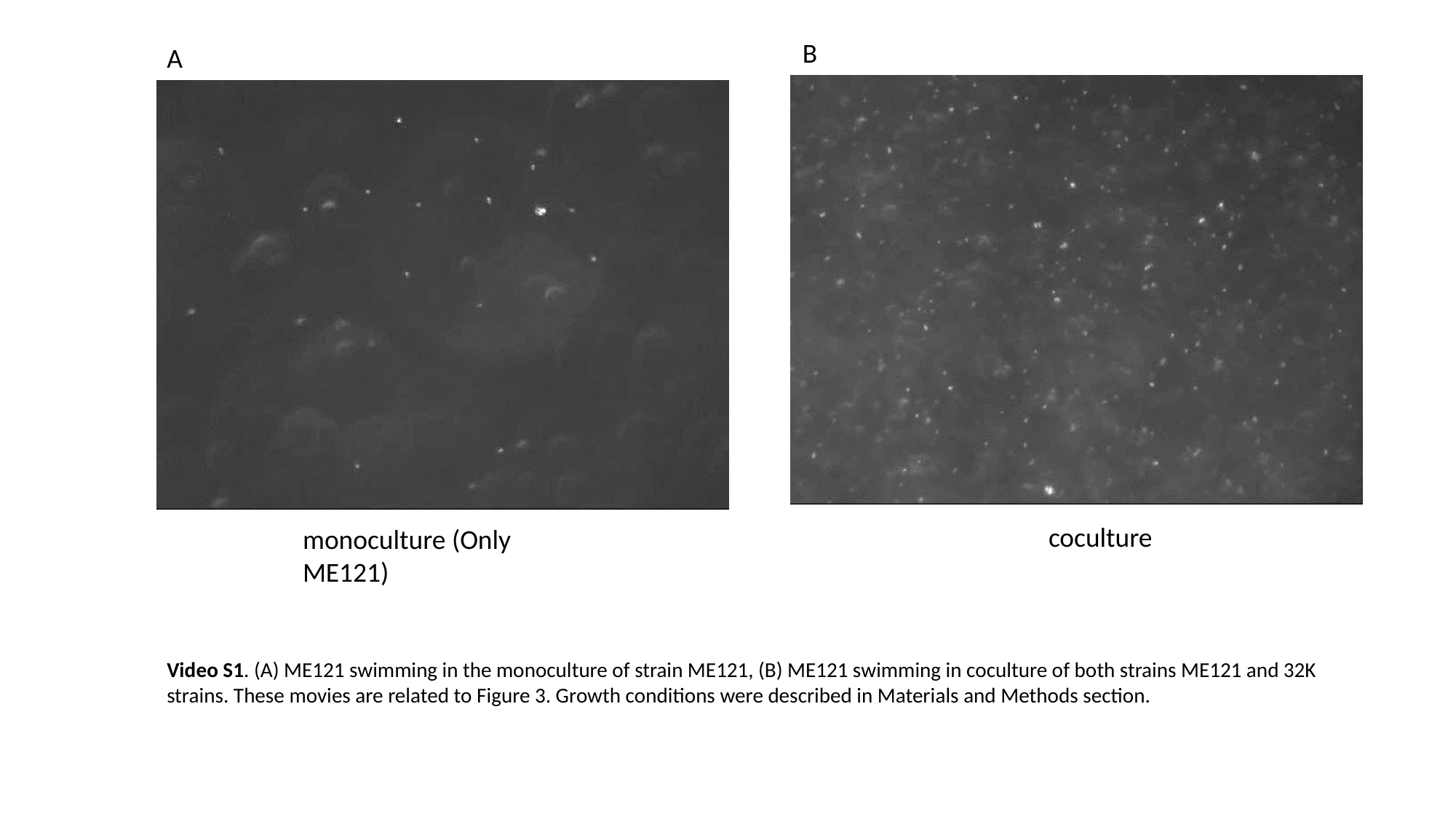

B
A
coculture
monoculture (Only ME121)
Video S1. (A) ME121 swimming in the monoculture of strain ME121, (B) ME121 swimming in coculture of both strains ME121 and 32K strains. These movies are related to Figure 3. Growth conditions were described in Materials and Methods section.

## Slide 2
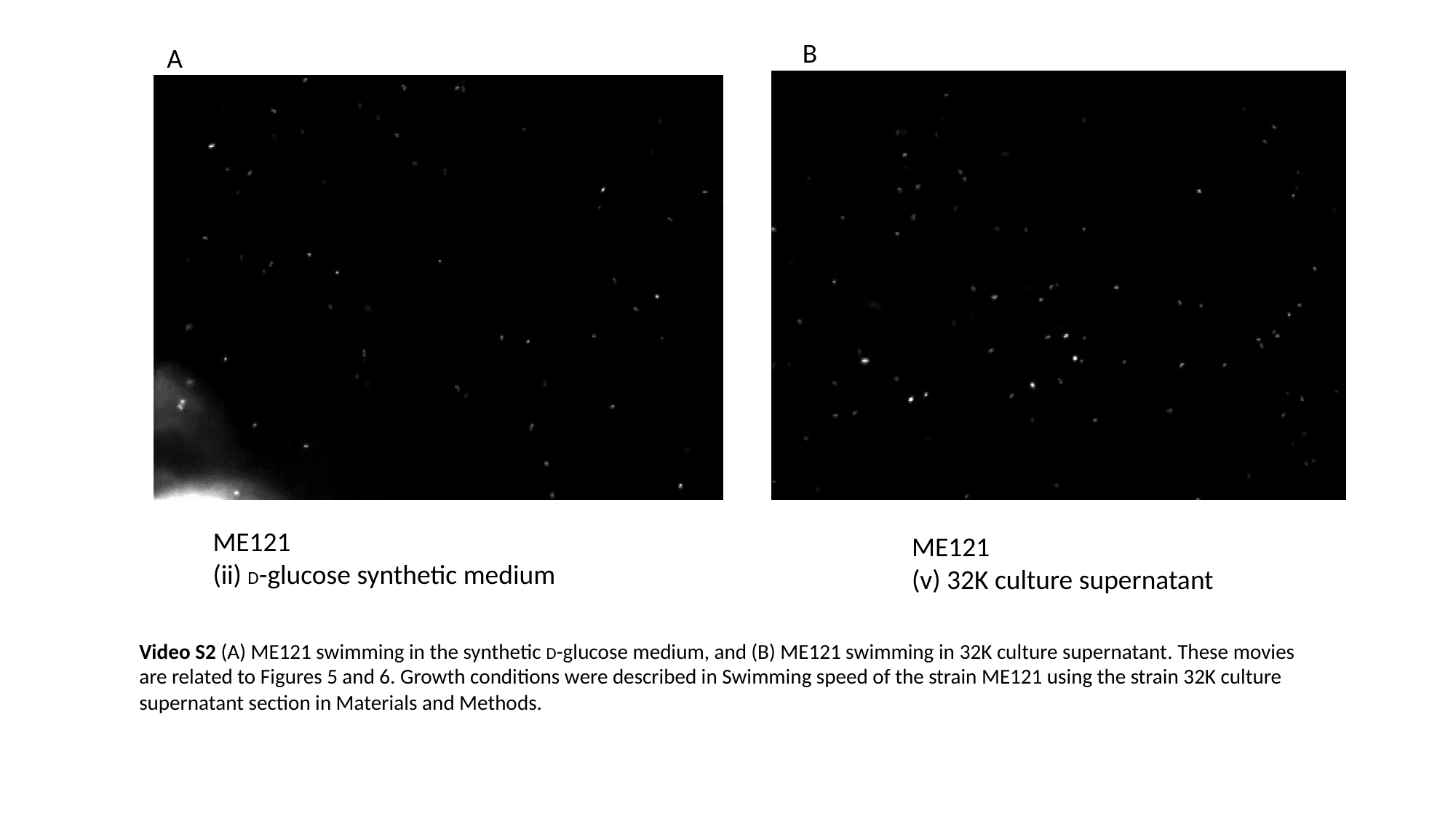

B
A
ME121
(ii) D-glucose synthetic medium
ME121
(v) 32K culture supernatant
Video S2 (A) ME121 swimming in the synthetic D-glucose medium, and (B) ME121 swimming in 32K culture supernatant. These movies are related to Figures 5 and 6. Growth conditions were described in Swimming speed of the strain ME121 using the strain 32K culture supernatant section in Materials and Methods.
